# Features of the cytoprotective effect of selenium nanoparticles on primary cortical neurons and astrocytes during oxygen–glucose deprivation and reoxygenation

**DOI:** 10.1038/s41598-022-05674-1

**Published:** 2022-02-02

**Authors:** E. A. Turovsky, V. N. Mal’tseva, R. M. Sarimov, A. V. Simakin, S. V. Gudkov, E. Y. Plotnikov

**Affiliations:** 1 Institute of Cell Biophysics of the Russian Academy of Sciences, Federal Research Center “Pushchino Scientific Center for Biological Research of the Russian Academy of Sciences”, Pushchino, Russia 142290; 2 Prokhorov General Physics Institute of the Russian Academy of Sciences, 38 Vavilove st., Moscow, Russia 119991; 3 A.N. Belozersky Institute of Physico-Chemical Biology, Lomonosov Moscow State University, Moscow, Russia 119992; 4 V.I. Kulakov National Medical Research Center of Obstetrics, Gynecology and Perinatology, Moscow, Russia 117997

**Keywords:** Biochemistry, Cell biology, Molecular biology, Neuroscience

## Abstract

The study is aimed at elucidating the effect of selenium nanoparticles (SeNPs) on the death of cells in the primary culture of mouse cerebral cortex during oxygen and glucose deprivation (OGD). A primary cell culture of the cerebral cortex containing neurons and astrocytes was subjected to OGD and reoxygenation to simulate cerebral ischemia-like conditions in vitro. To evaluate the neuroprotective effect of SeNPs, cortical astrocytes and neurons were incubated for 24 h with SeNPs, and then subjected to 2-h OGD, followed by 24-h reoxygenation. Vitality tests, fluorescence microscopy, and real-time PCR have shown that incubation of primary cultured neurons and astrocytes with SeNPs at concentrations of 2.5–10 µg/ml under physiological conditions has its own characteristics depending on the type of cells (astrocytes or neurons) and leads to a dose-dependent increase in apoptosis. At low concentration SeNPs (0.5 µg/ml), on the contrary, almost completely suppressed the processes of basic necrosis and apoptosis. Both high (5 µg/ml) and low (0.5 µg/ml) concentrations of SeNPs, added for 24 h to the cells of cerebral cortex, led to an increase in the expression level of genes Bcl-2, Bcl-xL, Socs3, while the expression of Bax was suppressed. Incubation of the cells with 0.5 µg/ml SeNPs led to a decrease in the expression of SelK and SelT. On the contrary, 5 µg/ml SeNPs caused an increase in the expression of SelK, SelN, SelT, SelP. In the ischemic model, after OGD/R, there was a significant death of brain cells by the type of necrosis and apoptosis. OGD/R also led to an increase in mRNA expression of the Bax, SelK, SelN, and SelT genes and suppression of the Bcl-2, Bcl-xL, Socs3, SelP genes. Pre-incubation of cell cultures with 0.5 and 2.5 µg/ml SeNPs led to almost complete inhibition of OGD/R-induced necrosis and greatly reduced apoptosis. Simultaneously with these processes we observed suppression of caspase-3 activation. We hypothesize that the mechanisms of the protective action of SeNPs involve the activation of signaling cascades recruiting nuclear factors Nrf2 and SOCS3/STAT3, as well as the activation of adaptive pathways of ESR signaling of stress arising during OGD and involving selenoproteins SelK and SelT, proteins of the Bcl-2 family ultimately leading to inactivation of caspase-3 and inhibition of apoptosis. Thus, our results demonstrate that SeNPs can act as neuroprotective agents in the treatment of ischemic brain injuries.

## Introduction

Cerebral ischemia is a common neurological disease that activates a cascade of pathophysiological events, including a decrease in oxygen and glucose levels, an excessive release of glutamate, a sharp increase in intracellular calcium concentration, and the release of free radicals. As a result, these events lead to disruption of homeostasis and functions of the endoplasmic reticulum and mitochondria, and, as a consequence, to the death of brain cells along the path of necrosis and/or apoptosis. In addition, the described pathophysiological events are accompanied by an inflammatory response that includes the activation of glial cells and the secretion of proinflammatory cytokines, which are believed to enhance apoptotic events leading to the loss of neurons^1–5^. Unfortunately, many neuroprotective drugs have failed to demonstrate efficacy in the treatment of ischemic stroke, since rapid metabolism and poor transport across the blood–brain barrier reduce the efficacy of most compounds^6^.

In connection with this problem, selenium is of great interest, playing an important role in ensuring the physiological activity of the nervous system, such as locomotor activity, coordination, memory, cognition, and signal transmission^7,8^. Selenium has been shown to participate in the pathophysiology of a number of neurodegenerative diseases, such as epilepsy, Alzheimer's disease and Parkinson's disease, where it is capable of exhibiting neuroprotective properties^9–13^. The neuroprotective effects of selenium are explained by its physicochemical properties, the ability to regulate oxidative stress, modulate Ca^2+^ influx through ion channels, and suppress apoptosis^14–16^. Besides, it was shown that selenium activates mitochondrial biogenesis signaling, protects the integrity of the mitochondrial structure, and reduces cerebral ischemic damage^17^.

The biological functions of selenium in the body are manifested mainly through 25 selenoproteins, in the active center of which is selenocysteine. Selenoproteins are largely expressed in the human brain. Their inherent antioxidant activity is provided by such antioxidant enzymes as glutathione peroxidase, thioredoxin reductase and selenoproteins P implicated in the protection system against free radicals and providing the maintenance of brain function^8^.

Of particular interest are selenium nanoparticles (SeNPs), which are low-dispersed bioactive compounds with powerful antioxidant capabilities, high bioavailability, and reduced toxicity compared to Se-containing compounds. SeNPs are able to cross the blood–brain barrier, accumulate in the brain and prevent the development of apoptosis in cells^18–21^. The uniqueness of nanoparticles is mediated by their properties—small size, high surface area, surface charge, surface chemistry, solubility and multi-functionality^22,23^. Nanoparticles are ideal for biomedical use because they are small enough (10–100 nm) not to be absorbed by the reticuloendothelial system, but large enough not to be filtered out in the kidneys^24–26^.

Thus, because of their promising therapeutic effect, SeNPs are actively used in studies aimed at studying the pathogenesis, prevention and/or treatment of various diseases, including neurodegenerative ones^27,28^. A number of investigations demonstrate that SeNPs affect the functional properties of hippocampal neurons, contribute to their survival regulating the antioxidant system, cellular metabolism, and inflammatory reactions accompanying ischemic damage. It is known that in the brain, SeNPs increase the expression of BDNF, decrease the level of β-amyloid and IL-6^29^. An increase in the level of BDNF expression contributes not only to the suppression of oxidative stress^30^, but also protects the most sensitive to hypoxia and ischemia GABAergic neurons from death^31,32^. In a mouse model of stroke, the authors reported that SeNPs were transferred to the brain via transferrin receptor-mediated endocytosis and were found to inhibit the inflammatory response and increase the survival of hippocampal neurons^3^. Despite the fact that the neuroprotective effects of SeNPs are widely represented, the mechanisms of the protective action, the effective concentrations of SeNP, features of the action of SeNPs on different populations of brain cells, both under physiological conditions and under pathological processes, as well as their effect on selenoproteins and their participation in neuroprotection remain unexplored.

Unfortunately, many neuroprotective drugs have failed to demonstrate efficacy in the treatment of acute ischemic stroke. The search for effective approaches and neuroprotective compounds is an urgent problem of modern biomedicine. It is known that antioxidants, anti-inflammatory cytokines, and receptor activators^32,33^ are able to suppress the global [Ca^2+^]_i_ increase in neurons and astrocytes.

However, the delivery of these compounds in the required effective concentration to the brain cells through the blood–brain barrier and the avoidance of the side effects of their high doses represent significant limiting factors for widespread clinical use.

Taking into account the above, the aim of this work was to elucidate the features of the action of Se NPs on primary cultured neurons and astrocytes in mice and to study the mechanism underlying the neuroprotective effect exerted by nanoparticles. The relationship of various concentrations of Se NPs with the induction/suppression of apoptosis and inhibition of necrosis, their effect on the expression of genes encoding ER selenoproteins and proteins regulating apoptosis in the cortical astrocytes and neurons was studied in detail when modeling ischemic conditions and reoxygenation.

## Materials and methods

All methods were carried out in accordance with relevant guidelines and regulations. Experimental protocols were approved by the Bioethics Committee of the Institute of Cell Biophysics. Experiments were carried out according to Act708n (23 August 2010) of the Russian Federation National Ministry of Public Health, which states the rules of laboratory practice for the care and use of laboratory animals, and the Council Directive 2010/63 EU of the European Parliament on the protection of animals used for scientific purposes.

### Animals

We used NMRI mouse line. Mice were kept in SPF cages 40 × 25 × 15 cm under standard laboratory conditions: a 12 h light circuit, 22 °C. Animals had free access to food and water. For housing, individually ventilated GM500 cages manufactured by Tecniplast (Italy) with a floor area of 500 cm^2^ were used. In the nests, the harem type of housing (2 females + 1 male) was carried out^34^. All animals were housing with littermates of the same sex in a group of 2–5 mice. All animal care and use protocols were conducted in accordance with the Standards of Humane Care and Use of Laboratory Animals of the Institute of Cell Biophysics, Puschino, Russia. All experiments involving animals also complied with the Guidelines for the Proper Conduct of Animal Experiments of the Bioethics Committee of the Institute of Cell Biophysics and Russian Federation National Ministry of Public Health (Act708n, 23 August 2010), and the ARRIVE guidelines for reporting animal research.

### Preparation and characterization of selenium nanoparticles

Selenium nanoparticles (SeNP) were obtained by laser ablation in deionized water. The solid target was placed at the bottom of a cuvette under a thin layer of water. In this state, the solid target was irradiated with a laser beam (λ = 1064 nm; *T* = 4–200 ns; *f* = 20 kHz; *P* = 20 W; *E*_*p*_ = 1 mJ). The laser beam was mixed on the target using a galvanomechanical scanner TM 2D (Ateko, Russia). Depending on the characteristics of the laser radiation, the speed and trajectory of the laser beam, it is possible to obtain colloidal solutions of selenium nanoparticles with specified geometric parameters. The procedure for obtaining and characterization selenium nanoparticles was described in detail earlier^34^. The nanoparticle size was characterized using a DC24000 analytical centrifuge (CPS Instruments, USA). The nanoparticle concentration and hydrodynamic radius were evaluated using the Zetasizer Ultra Red Label (Malvern, UK). The morphology of the nanoparticles was studied by electron energy loss spectroscopy using a 200FE transmission electron microscope (Carl Zeiss, Germany).

**Figure 1:**
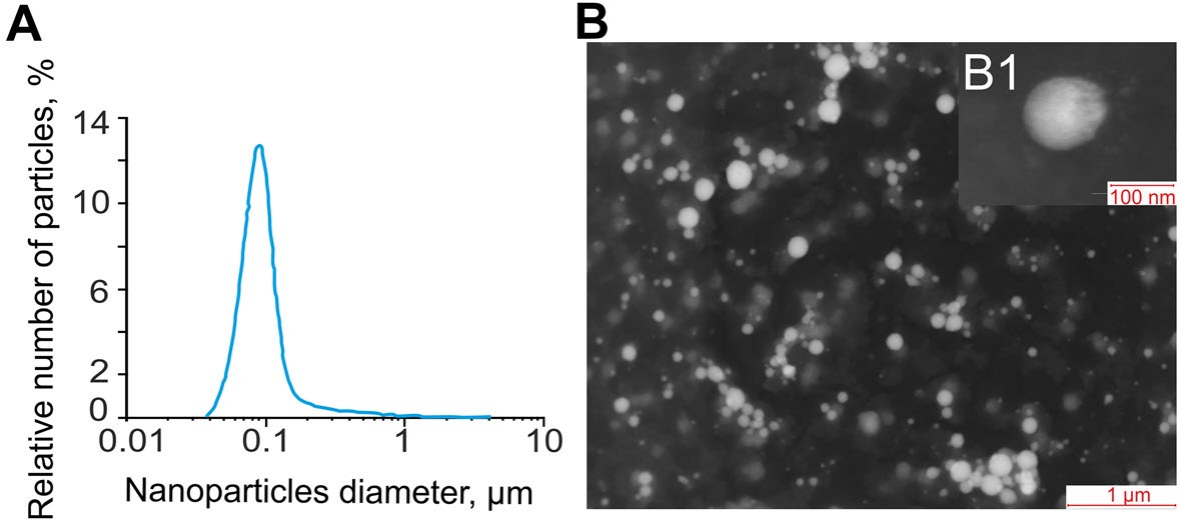
Size and morphology of selenium nanoparticles obtained by laser ablation. (**A**) Selenium nanoparticle size distribution. Data obtained using an analytical disk centrifuge and confirmed by DLS. (**B**) TEM micrograph of selenium nanoparticles. Scale bar—1 μm. (**B1**) TEM micrograph of single selenium nanoparticle.

The nanoparticles size distribution was investigated using a disk analytical centrifuge (Fig. 1A). As we have shown earlier^34^ the obtained preparation of selenium nanoparticles has a monomodal size distribution. The average nanoparticle size is about 0.1 µm, the half-width is in the range 0.075–0.125 µm. The results obtained were confirmed by dynamic light scattering. The concentration of nanoparticles in the colloid was investigated using a Zetasizer Ultra Red Label; it was shown that, at a concentration of up to 10^13^ nanoparticles per ml, there is no aggregation in the colloidal solution. Aggregation was not detected during storage of an aqueous colloidal solution of selenium nanoparticles for a month. The zeta potential of the colloidal solution was 16.2 mV. The transmission electron microscope image showed Se nanoparticles with a diameter of about 0.1 µm (Fig. 1B). Particle sizes shown in TEM photomicrographs matched the size distribution plotted using an analytical disk centrifuge. The shape of the nanoparticles is spherical (Fig. 1B1).

### Preparation of mixed cortical neuronal and glial cell cultures

All procedures were approved by the Animal Ethic committee of the Institute of cell biophysics. Cell cultures were prepared as described in detail previously^36^. Briefly, 0–1 day mice (NMRI mouse line) were decapitated after a brief (45–60 s) anesthesia with carbon dioxide before sacrifice. Mice were subjected to cervical dislocation and disinfected with 70% ethanol prior to dissection. The extracted cortex was washed with Mg^2+^- and Ca^2+^-free Versene solution and minced with scissors. Then, the tissue fragments were digested with 1% trypsin solution for 10 min at 37 °C and washed two times with cold Neurobasal-A medium. Trypsinized tissue was gently triturated with a pipette, and the debris was then carefully removed with a pipette tip. The obtained cell suspension was seeded on polyethyleneimine-coated glass coverslips and grew for 10 days in vitro in the cell culture medium composed of Neurobasal-A medium, supplement B-27 (2%) and 0.5 mM glutamine. On average, a suspension with a cell concentration of 2.5 ± 1.5 million/ml was obtained from a piece of the mouse cerebral cortex. A 250 ± 50 thousand cells were placed on each coverslips. On the 10th day in vitro cultivation, 100 ± 50 thousand cells were found on the coverslips, since when cells were isolated using trypsin, 30–40% of the cells in suspension did not survive in the first days of cultivation.

The drugs and SeNPs were added into culture medium under sterile conditions in the case of experiments with 24-h pre-incubation. Then, the cell cultures were washed after the pre-incubation with Hank`s balanced salt solution and used in experiments.

### The technique for simulation of ischemia-like conditions

Ischemia-like conditions (oxygen–glucose deprivation, OGD) were obtained by omitting glucose (HBSS medium without glucose) and by displacement of dissolved oxygen with argon in the leak-proof system^37^. The level of oxygen in the medium was measured using a Clark electrode. Oxygen tensions reached values 30–40 mm Hg or less within 20 min after the beginning of displacement. Ischemia-like conditions lasting for 40 min were created using supplying the oxygen–glucose deprivation (OGD)-medium into the chamber with cultured primary cultured neurons and astrocytes. Constant argon feed into the experimental chamber was used to prevent the contact of the OGD-medium with the atmospheric air.

### Assessment of cell viability and apoptosis

Hoechst 33342 (2 µM) and propidium iodide (1 µM) were used to evaluate the number of dead cells in the cell cultures before and after OGD. The cells were stained for 5 min with the probes diluted in HBSS and then rinsed with HBSS. Fluorescence of the probes was detected with an inverted fluorescent microscope Zeiss Axio Observer Z1 using Filter Set 01 and Filter Set 20. Discrimination of early and late apoptotic cells was performed according to the previously described method^31,38^. It is known that viable cells are not permeable to PI, while Hoechst 33342 penetrates through the plasma membrane, staining the chromatin. Under our conditions of cultivation, cell proliferation does not occur and primary cultured neurons and astrocytes were defined as apoptotic if the intensity of Hoechst 33342 fluorescence was 3–4 times higher compared to Hoechst 33342 fluorescence in healthy cells, indicating chromatin condensation, which can occur as a result of apoptosis induction. The differences between the early and late stages of apoptosis were determined by the intensity of Hoechst 33342 fluorescence, and at the later stages of apoptosis, cells begin to show insignificant membrane permeability for PI. Five different areas of each cell culture were analyzed. Each experimental group consisted of three five cultures from different passages and from each passage, 3 coverslips with cells were taken into the each experimental group.

To simultaneously monitor apoptotic and healthy cells after SeNP treatment or SeNP + OGD/R with fluorescence microscope, Apoptosis/Necrosis Detection Kit was used^39^. Cells were resuspended with Assay Buffer. To detect apoptotic cells, Apopxin Green Indicator was used. Apoptotic cells were visualized using the FITC channel (λ_ex_/λ_em_ = 490/525 nm). For staining necrotic cells, we used 7‐aminoactinomycin D (λ_ex_/λ_em_ = 550/650 nm). To detect healthy cells, CytoCalcein 450 was used and cells were visualized using the violet channel (λ_ex_/λ_em_ = 405/450 nm). To examine the effect of SeNPs on OGD-induced initiation of apoptosis, a fluorescent probe, NucView488 caspase-3 substrate, was used for staining^40^. In this regard, before the experiments, the cultures were loaded with NucView488 for 1 h (final concentration of 2 μm). The cultures were then subjected to 40-min oxygen–glucose deprivation and 2-h reoxygenation. NucView488 fluorescence was recorded using an image analysis system based on an Axiovert 200M inverted fluorescence microscope equipped with a Hamamatsu ORCA-Flash 2.8 high-speed monochrome CCD camera. A Lambda DG-4 Plus illuminator (Sutter Instruments, USA) with a high-pressure mercury lamp was used. To excite and register the NucView488 emission, we used a set of light filters: Filter Set 10 with excitation filter BP 450–490, beam splitter FT510, emission filter BP 515–565. Each experimental series consisted of at least three separate repeats.

To distinguish neurons and astrocytes, we used short-term applications of 35 mM KCl and 10 µM ATP before the main experiments. This method was described in detail in our previous work^4^. Briefly, KCl induces depolarization of excitable cells, which contain a wide range of voltage-gated cation channels. KCl-induced depolarization promotes the opening of voltage-gated calcium channels in neurons (predominantly L-type channels). The conductivity and density of cation channels in astrocytes are insufficient to evoke high-amplitude Ca^2+^-response to KCl application.

### Extraction of RNA

As in our previous works^39,40^ Mag Jet RNA Kit (Thermo Fisher Scientific, USA) was used for the extraction of total RNA. The RNA quality was estimated by electrophoresis in the presence of 1 μg/ml ethidium bromide (2% agarose gel in Tris/Borate/EDTA buffer). The concentration of the extracted RNA was determined with NanoDrop 1000c spectrophotometer. RevertAid H Minus First Strand cDNA Synthesis Kit (Thermo Fisher Scientific, USA) was used for reverse transcription of total RNA.

### Real-time polymerase chain reaction (RT-qPCR)

Each PCR was performed in a 25 μL mixture composed of 5 μL of qPCRmix-HS SYBR (Evrogen, Moscow, Russia), 1 μL (0.2 μM) of the primer mix, 17 μL water (RNase-free), 1 μL cDNA. Dtlite 5 Real-Time PCR System (DNA-technology, Moscow, Russia) was used for amplification. Amplification process consisted of the initial 5 min denaturation at 95 °C, 40 cycles of 30 s denaturation at 95 °C, 20 s annealing at 60–62 °C, and 20 s extension step at 72 °C. The final extension was performed for 10 min at 72 °C. The sequences of the used primers are presented in Table 1. All the sequences were designed with FAST PCR 5.4 and NCBI Primer-BLAST software. The data were analyzed with Dtlite 5 software (DNA-technology, Moscow, Russia). The expression of the studied genes was normalized to gene encoding Glyceraldehyde 3-phosphate dehydrogenase (GAPDH). Data were analyzed using Livak’s method^41^.

**Table 1:** Primer sequences for real-time polymerase chain reaction (RT-PCR).

Gapdh	Forward 5′-tccactcacggcaaattcaac-3′
	Reverse 5′-cggcatcgaaggtggaagag-3′
Bcl-2	Forward 5′-ctacgagtgggatgctggagatg-3′
	Reverse 5′-tcaggctggaaggagaagatgc-3′
Bax	Forward 5′-taaagtgcccgagctgatcagaac-3′
	Reverse 5′-cttcccagccaccctggtctt-3′
Bcl-xL	Forward 5′-tggccacagcagcagtttg-3′
	Reverse 5′-tctccggtaccgcagttcaa-3′
Stat3	Forward 5′-ttctgggcacgaacacaaaagt-3′
	Reverse 5′-gcctccattcccacatctctg-3′
Socs3	Forward 5′-aagaacctacgcatccagtgtga-3′
	Reverse 5′-atgtagtggtgcaccagcttgag-3′
Nrf2	Forward 5′-tcctggacgggactattgaaggctg-3′
	Reverse 5′-cacattgggattcacgcataggagcact-3′
SelK	Forward 5′-atctcgaatggtcaggtgttg-3′
	Reverse 5′-accttcctcatccaccagc-3′
SelN	Forward 5′-ctcggtgccctctgtgatc-3′
	Reverse 5′-gggctttccaggacagtctc-3′
SelT	Forward 5′-agacatccgcattgaaggc-3′
	Reverse 5′-agttgttgcatggatggaag-3′
SelP	Forward 5′-aggagaggtgcggaaactg-3′
	Reverse 5′-tcctctgggcaagtgaaag-3′
Eif2ak1	Forward 5′-agatatgtatagcttgggtgtg-3′
	Reverse 5′-atttccttctcttgttctattatc 3′
MLKL	Forward 5′-caaagagcactaaagcagagag-3′
	Reverse 5′-ggcaatcctgacccactgg 3′
RIP1	Forward 5′-aaggagccctatgagaatgtc-3′
	Reverse 5′-acatcctcttctacatattcttc 3′
TRAIL	Forward 5′-ctaaccacaacacggaacctg-3′
	Reverse 5′-cagcagatggttgatggaggc 3′
Cas-1	Forward 5′-ttattcaggcatgccgtggag-3′
	Reverse 5′-tcctccaagtcacaagaccag 3′

### Statistical analysis

All presented data were obtained from at least three cell cultures from 3 different passages. All values are given as mean ± standard error (SEM). PCR analysis was performed and analyzed as blinded, the experimenter was unaware of the concentrations of SeNPs. Experiments using fluorescence microscopy were performed with a significant number of replicates and their statistical processing provides significant differences between the experimental groups. Statistical analyses were performed by one-way ANOVA followed by the Tukey–Kramer test. MS Excel, ImageJ, Origin 2016 (OriginLab, Northampton, MA, USA), and Prism GraphPad 7 (GraphPad Software, RRID: SCR_002798) software was used for data and statistical analysis^40^.

### Ethical approval

All animal procedures were fulfilled in accordance with the experimental protocols were approved by the Bioethics Committee of the Institute of Cell Biophysics. Experiments were carried out according to Act708n (23 August 2010) of the Russian Federation National Ministry of Public Health, which states the rules of laboratory practice for the care and use of laboratory animals, and the Council Directive 2010/63 EU of the European Parliament on the protection of animals used for scientific purposes.

## Results

### Dose-dependent effect of SeNPs on apoptosis and necrosis in mouse cortical cells

**Figure 2:**
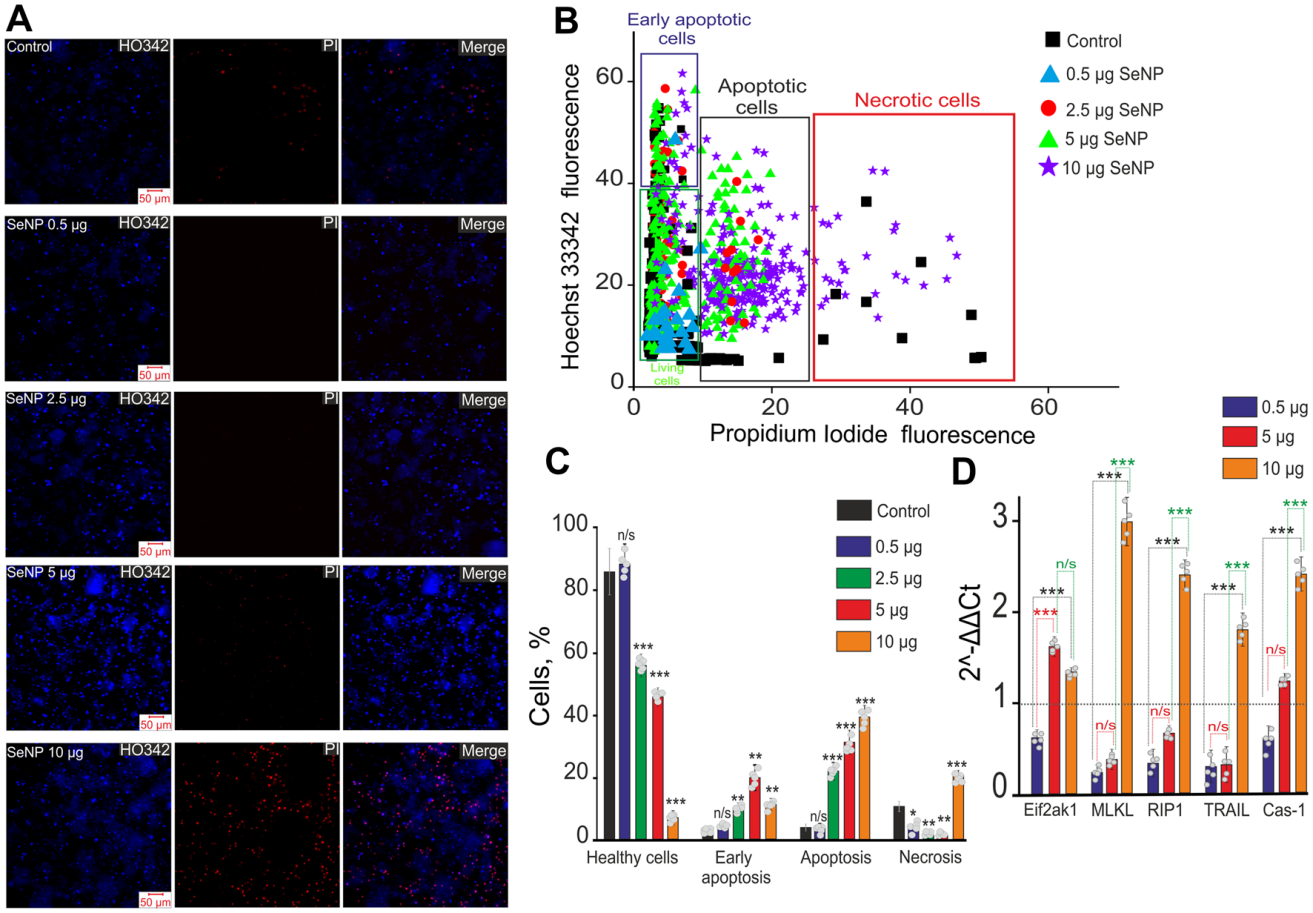
The action of different concentrations of SeNPs on induction of apoptosis and necrosis in the cortical cells. (**A**) Double staining of cells with Hoechst 33342 (HO342), Propidium iodide (PI) and merge (Merge) between Hoechst342 and PI 24 h after SeNPs application. (**B**) Cytogram demonstrating the viability of cortical primary cultured neurons and astrocytes. X-axis—the intensity of PI fluorescence; Y-axis—the intensity of Hoechst 33342 fluorescence. Cells were stained with the probes 24 h after the SeNPs application. (**C**) Effects of different SeNPs concentrations on the induction of necrosis and apoptosis 24 h after SeNPs applications. The percentage of living cells (black column) and cells in which the processes of early apoptosis (violet column), apoptosis (green column), and necrosis (red column) were detected. Each point represents the mean from an individual cell culture. At least 2000 cells were analyzed for each sample. N cell cultures = 5; n coverslips with cells for each sample = 15. Statistical significance was assessed using one-way ANOVA followed by the Tukey–Kramer test. (**D**) Effect of different concentrations of SeNPs on the level of expression of genes encoding marker proteins for necrosis induction. Analyzed 5 samples (5 cell cultures) for each concentration of SeNPs. The horizontal line corresponds to the expression of mRNA of the studied genes in control samples (without SeNPs application). Statistical significance was assessed using one-way ANOVA followed by the Tukey–Kramer test. All values are reliable relative to control (***P < 0.001). For panels (**C**) and (**D**), results are presented as mean ± SEM. n/s—data not significant (*p* > 0.05), *P < 0.05, **P < 0.01, and ***P < 0.001 compared experimental groups (+ SeNPs) with Control (without SeNPs).

There is evidence about the dual effects of selenium on the development of apoptosis^3,42,43^. Since we investigated the neuroprotective properties of SeNPs on brain cells, we decided to test the effect of different concentrations of SeNPs on cell survival. With the application of different concentrations of SeNPs (2.5–10 µg/ml), we observed a decrease in the number of living cells. In the control, during the cultivation and development of the cellular network (10 DIV), death by the necrosis occurred in 7 ± 4% of cells and about 5 ± 2% of cells at various stages of apoptosis (Fig. 2, Control), which was recorded by the presence of PI fluorescence and HO342 (Fig. 2A,B). Application of SeNPs to cultured cells of the mouse cerebral cortex (10 DIV) for 24 h at concentrations of 2.5, 5, and 10 μg/ml dose-dependently induced apoptosis, which was recorded by an increased level of HO342 fluorescence and the appearance of PI fluorescence (Fig. 2A,B). Incubation of cells for 24 h with 2.5 µg/ml SeNPs leads to an increase in the number of cells at the early and late stages of apoptosis to 27 ± 6% and 11 ± 8%, respectively (Fig. 2B,C). Interestingly, the number of necrotic cells in culture at this concentration of SeNPs decreases to 3 ± 2% (Fig. 2A–C). An increase in the concentration of SeNPs to 5 μg/ml leads to an increase in the number of cells at the early and late stages of apoptosis to 21 ± 11% and 34 ± 7%, occurring against the background of reduced cell death by the necrosis (by 2 ± 2%), respectively (Fig. 2A–C). At the same time, 10 µg/ml SeNPs lead to the activation of necrosis (Fig. 2A, SeNP 10 µg) in the neurons and astrocytes of the cerebral cortex—28 ± 8% with increase in the number of cells at the late stage of apoptosis—51 ± 14% (Fig. 2B,C). The effect of the low concentration of SeNPs (0.5 µg/ml) is of interest. The number of surviving cells increases, there is a complete inhibition of basic necrosis in culture (Fig. 2A, the absence of cells with PI fluorescence, SeNP 0.5 µg is shown) and suppression of apoptosis (single cells at an early stage of apoptosis, Fig. 2B) and in the late stages of apoptosis is no more than 6 ± 4% of cells (Fig. 2B,C). Additional experiments using the Apoptosis/Necrosis Detection Kit confirmed that preincubation of cells with 5 µg/ml SeNPs led to the induction of apoptosis without a significant increase in necrotic cells, but an increase in the concentration of SeNPs up to 10 µg was accompanied by the activation of necrosis (Supplementary, S1. Fig. 1). For a more detailed study of the induction of the process of necrosis in cells, a PCR analysis of the expression of key necrosis marker genes was performed (Fig. 2D). Pre-incubation of cells with 0.5 µg/ml SeNPs resulted in a decrease in the expression of all 5 studied genes encoding necrosis marker proteins (Fig. 2D, purple bars). An increase in the concentration of SeNPs to 5 µg/ml led to an increase in the expression of the Eif2ak1 and Cas-1 genes, against the background of a decrease in MLKL, RIP1 and TRAIL (Fig. 2D, red bars), which, together with the results of vitality tests, may indicate some induction of necrosis. However, 10 µg/ml SeNPs resulted in increased expression of all 5 genes encoding necrosis proteins, suggesting the toxicity of this dose of selenium nanoparticles (Fig. 2D, orange bars).

Thus, incubation of cortical cell cultures with selenium nanoparticles at concentrations of 2.5–10 µg/ml leads to a dose-dependent activation of the apoptosis process, while a low concentration of SeNPs—0.5 µg/ml, on the contrary, almost completely suppresses basic necrosis and apoptosis, which may indicate about the neuroprotective properties of SeNPs. Interestingly, against the background of an increase in the number of cells at various stages of apoptosis at high concentrations of SeNPs (2.5–5 µg/ml), the suppression of basic necrosis in culture also occurs.

### Dose-dependent activation of caspase-3 by selenium nanoparticles in neurons and astrocytes of the brain. Changes in the expression of selenoprotein genes and apoptosis markers

**Figure 3:**
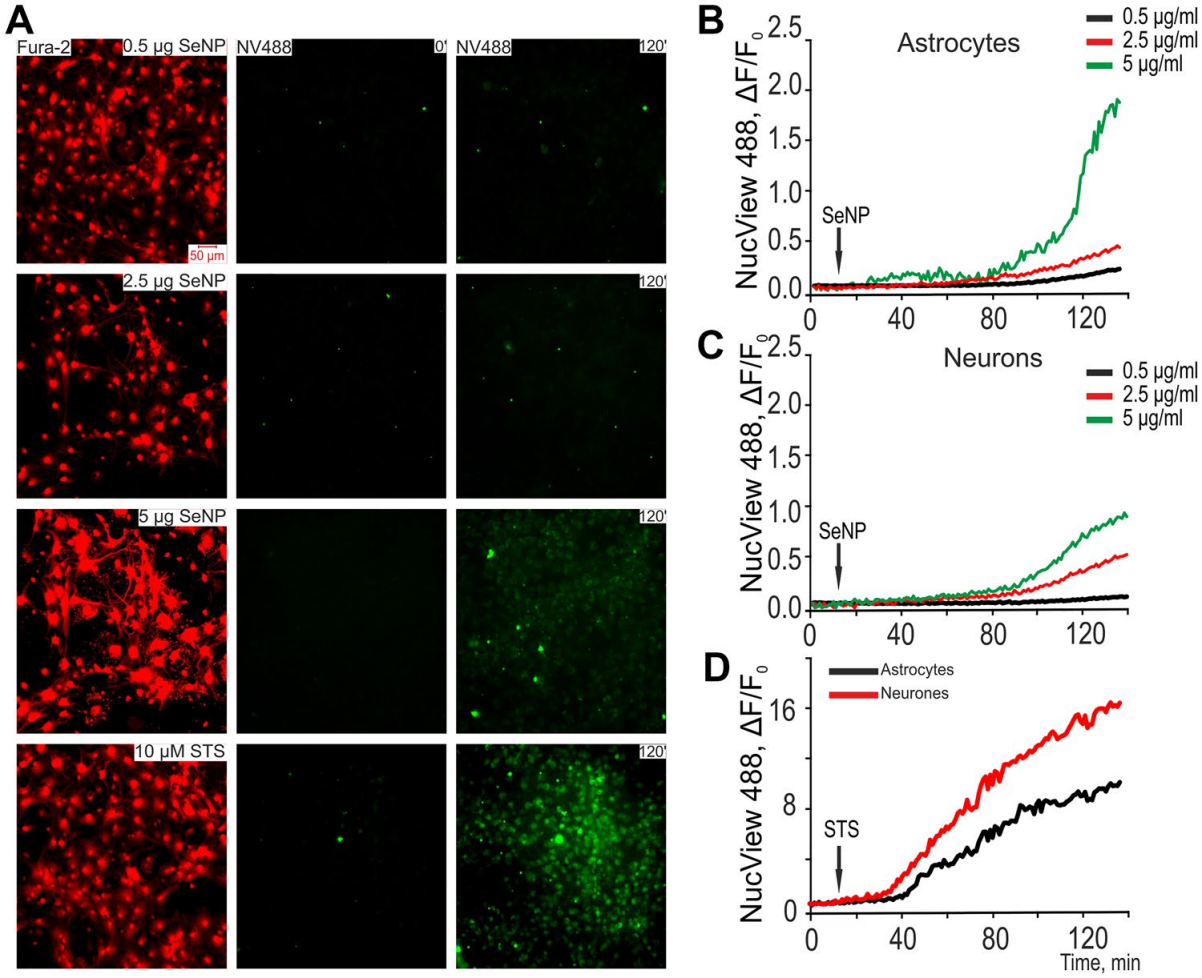
Effect of SeNPs on the activation of caspase-3-induced apoptosis in astrocytes and neurons of the cerebral cortex in vitro. (**A**) Images of cortical cell cultures loaded with a fluorogenic substrate of caspase-3, NucView-488, which were exposed to different concentrations of SeNPs and staurosporine (STS) within 120 min. Images of the cell culture in NucView-488 fluorescence detection channel before experiments (0′) and after 120-min SeNPs treatment are represented. Images correspond to the curves shown in panels (**B**), (**C**) and (**D**) (for STS). The appearance of green color in the micrograph means the appearance of NucView-488 fluorescence, which indicates the activation of Caspase-3 and apoptosis. (**B**, **C**) Hydrolysis of the fluorogenic substrate of caspase-3 (NucView-488) during a SeNPs treatment, indicating apoptosis induction in cortical astrocytes (**B**) and neurons (**C**). (**D**) Hydrolysis of the fluorogenic substrate of caspase-3 (NucView-488) during a 10 µM staurosporine treatment, indicating apoptosis induction in cortical astrocytes (black) and neurons (red). We used objective HCX PL APO 20.0 × 0.70 IMM UV, refraction index 1.52. Camera settings is 500 pixels × 500 pixels (Voxel Size 0.724 µm × 0.723 µm), binning 2 × 2, resolution 14 bits. There were 110 ± 87 cells in the field of view of the objective, as indicated by Fura-2 fluorescence in panel (**A**) (red, 380 nm channel).

In the experiments, we used mixed cell cultures of the mouse cerebral cortex. To identify neurons and astrocytes, their Ca^2+^ signals were used for the application of KCl and ATP, for which the cells were loaded with a Ca^2+^-sensitive Fura-2 probe (Fig. 3A). After identifying the cell type, the fluorescence of NucView-488 was recorded. The ratio of neurons to astrocytes in the used cell cultures, revealed by staining with antibodies against GFAP, averaged 37 ± 18% of astrocytes and 74 ± 15% of neurons (Supplementary, S1 Fig. 2). Control experiments (Supplementary, S1 Fig. 3) showed that short-term applications of KCl and ATP to cells did not affect the rate of SeNPs-induced activation of caspase-3, recorded by NucView-488 fluorescence.

Since we observed an increase in the number of cells in apoptosis when exposed to all the used concentrations of SeNPs, we decided to analyze the level of activation of caspase-3 when exposed to these concentrations. The activation of the terminal caspase cascade of caspase-3 is a marker of the induction of apoptosis in cells. Cell cultures were pre-incubated for 1 h with the fluorogenic substrate of caspase-3, NucView-488. Almost no fluorescence of NucView-488 was observed prior to application of SeNPs (not shown). Then, various concentrations of SeNPs were added to the cells, and the fluorescence of NucView-488 was recorded for 140 min at an interval of 1 frame every 60 s. Application of 0.5 μg/ml SeNPs led to the appearance of NucView-488 fluorescence in single cells (Fig. 3A) and these were astrocytes (Fig. 3B), whereas in the neuronal population, caspase-3 activation did not occur even after 120 min of measuring fluorescence (Fig. 3C). The application of 2.5 µg/ml SeNPs causes the activation of caspase-3 after an average of 80 ± 10 min in 10–15% of the cells in the field of view of the microscope (Fig. 3A). Moreover, the rates of caspase-3 activation in response to 2.5 µg/ml SeNPs in astrocytes (Fig. 3B) and neurons (Fig. 3C) are approximately the same and amount to 0.005 ± 1.3^−4^ and 0.006 ± 2^−4^, respectively (Table 2).

**Table 2:** The rate of increase in fluorescence of NucView-488 caspase-3 substrate (± SE), reflecting the induction of apoptosis.

	SeNPs			OGD 40 min		Reox 2 h		STS
	0.5 µg	2.5 µg	5 µg	w/o	0.5 µg	w/o	0.5 µg	10 µM
Astrocytes	0.0023 ± 9.5^−5^	0.005 ± 1.3^−4^	0.037 ± 0.002	0.023 ± 0.002	3.99^−4^ ± 6.6^−5^	0.04 ± 0.003	0.03 ± 4.8^−4^	0.01 ± 0.002
Neurons	0.005 ± 4.5^−5^	0.006 ± 2^−4^	0.02 ± 2.9^−4^	0.03 ± 0.002	5.52^−4^ ± 9.3^−5^	0.07 ± 0.002	0.06 ± 4.8^−4^	0.16 ± 0.003

For each group, 12 samples from 5 cell cultures with cells were analyzed.

An increase in the concentration of SeNPs to 5 μg/ml leads to the induction of apoptosis in 30–40% of cells (Fig. 3A) and a slight increase in NucView-488 fluorescence in astrocytes occurs already after 20 ± 15 min with an exponential increase after 70 min (Fig. 3B). In neurons, this process is less pronounced and a slower increase in the production of caspase-3 is observed after 80 min (Fig. 3C). A more pronounced effect of 5 µg/ml SeNPs on astrocytes is confirmed by measuring the rate of activation of caspase-3, which averages 0.037 ± 0.002, compared with neurons—0.02 ± 2.9^−4^ (Table 2).

Interestingly, the apoptosis inducer staurosporine (Fig. 3A, STS, 10 µM), added to the cortical culture, causes an increase in NucView-488 fluorescence in 60–75% of cells (Fig. 3D) at a higher rate in neurons—0.16 ± 0.003 (Fig. 3D, red curve; Table 2), compared with astrocytes—0.01 ± 0.002 (Fig. 3D, black curve; Table 2).

**Figure 4:**
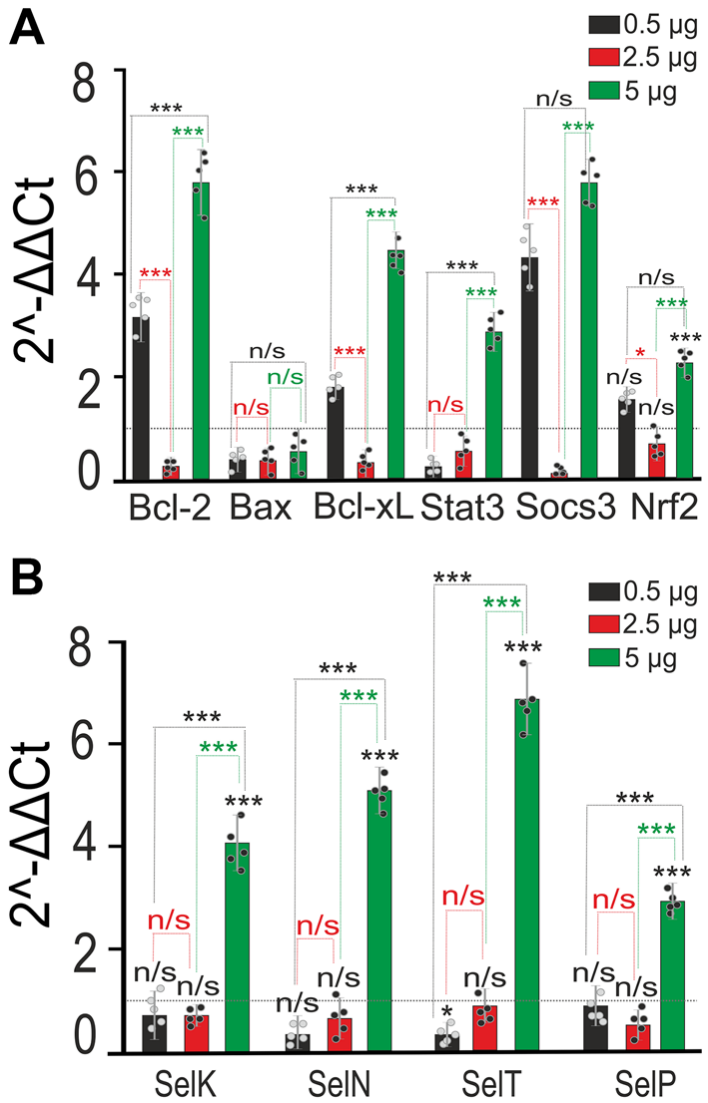
Effect of SeNPs on the basal expression of genes, encoding pro-apoptotic proteins and selenoproteins. (**A, B**) Effect of 24-h incubation of cortical cells with 0.5 and 5 µg/ml SeNPs on basal expression of genes involved in regulation of apoptosis (**A**) and coding of selenoproteins (**B**). The number of samples is 5 (5 cell cultures). Dashed line level of gene expression in controls (without SeNPs). Statistical significance was assessed using one-way ANOVA followed by the Tukey–Kramer test. n/s—data not significant (*p* > 0.05), *P < 0.05, **P < 0.01, and ***P < 0.001.

To analyze the participation of apoptosis marker genes in the activation of caspase-3, we selected two concentrations of SeNPs—0.5 and 5 µg/ml. Both high (5 µg/ml) and low (0.5 µg/ml) concentrations of SeNPs, added for 24 h to the cells, lead to an increase in the expression of genes encoding anti-apoptotic proteins—Bcl-2, Bcl-xL, and inflammatory Socs3. But the expression of the pro-apoptotic gene, Bax, on the contrary, is suppressed (Fig. 4A). After incubation of cells with 0.5 μg/ml, there is an increase in the expression of genes Bcl-2, Bcl-xL, Socs3 by 73%, 2.9 times, 71% and 4.9 times, compared with the control (Fig. 4A, dashed line, taken for 1), and the expression of Bax and Stat3 is suppressed by 61% and 97%, respectively. At the same time, 5 µg/ml SeNPs increase the expression of Bcl-2, Bcl-xL, Stat3 and Socs3 by 3.4, 5.87, 4.11, 2.3 and 6.2 times, respectively (Fig. 4A, red bars). The expression level of the gene encoding Nrf 2 also tends to increase after incubation with both concentrations of SeNPs.

Incubation of cells with SeNPs alters the level of selenoprotein expression (Fig. 4B). A low concentration of SeNPs (0.5 µg/ml) leads after 24 h of incubation to a trend towards a decrease in the genes SelK (by 47%) and SelT (by 98%), while the expression of SelN and SelP does not significantly decrease (Fig. 4B, black bars). A high concentration of SeNPs (5 µg/ml), on the contrary, causes an increase in the expression of SelK, SelN, SelT, SelP by 3.82, 4.21, 6.4 and 2.37 times, respectively (Fig. 4B, red bars).

Thus, high concentrations of SeNPs 2.5–5 µg/ml lead to the induction of apoptosis through the activation of caspase-3 with a higher rate of increase in the process in astrocytes as compared to neurons. Both low and high concentrations of SeNPs cause an increase in the expression of genes encoding anti-apoptotic proteins and a decrease in the level of pro-apoptotic (Bax), which indicates the activation of apoptosis and neuroprotective signaling cascade along with the caspase-signaling pathway. However, the level of genes expression encoding selenoproteins after incubation with 0.5 µg/ml SeNPs decreases, and at 5 µg/ml, on the contrary, increases, which may indicate the suppression of endoplasmic reticulum stress, in the case of low doses, and activation of this process at using high doses.

### Protective action of SeNPs at oxygen–glucose deprivation/reoxygenation in vitro. Changes in expression of genes encoding selenoproteins and pro-apoptotic genes

**Figure 5:**
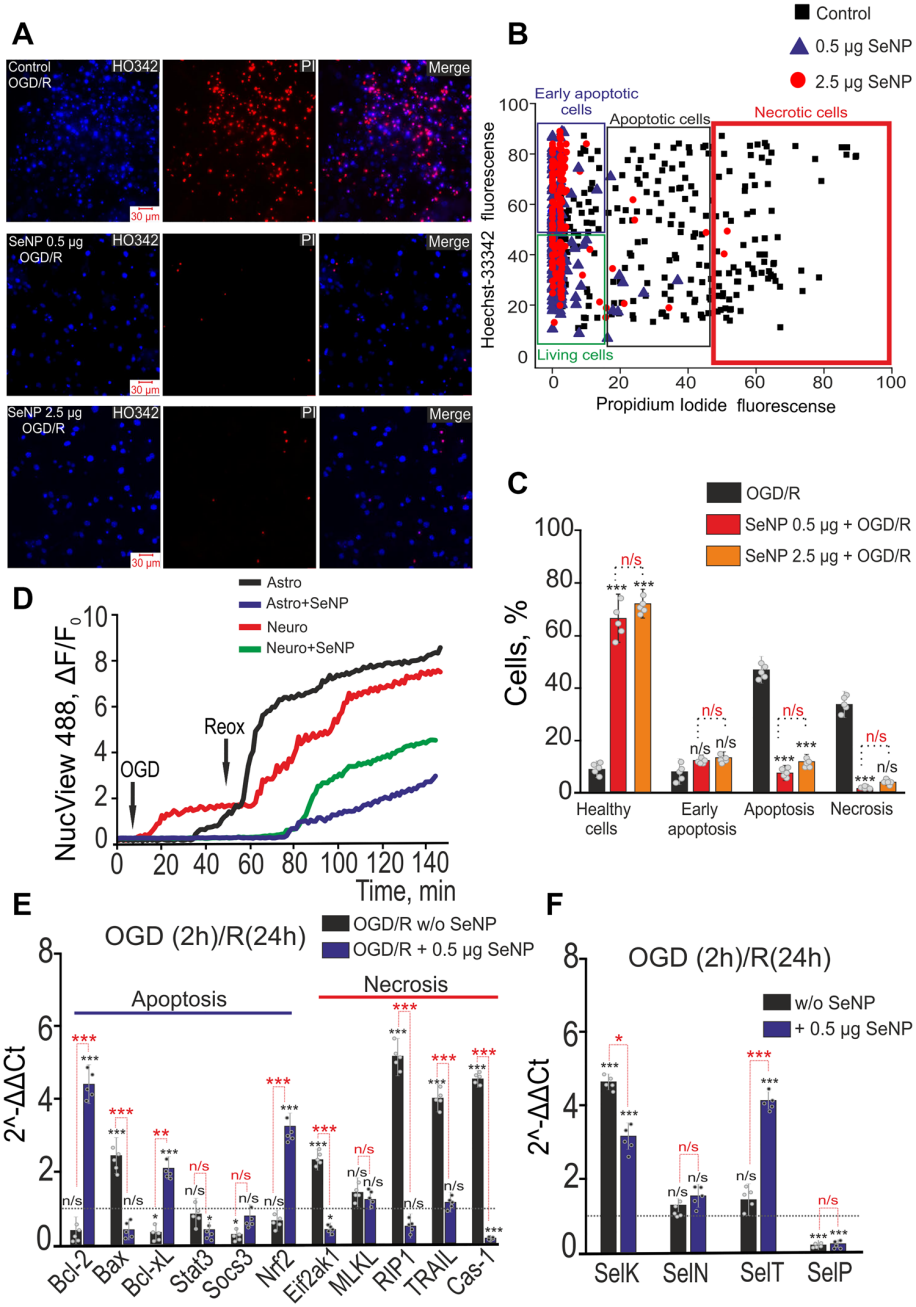
Anti-apoptotic role of SeNPs under OGD and during reoxygenation (R). (**A**) Double staining of cells with Hoechst 33342 (HO342), Propidium iodide (PI) and merge (Merge) between Hoechst342 and PI after 2 h OGD and 24 h reoxygenation (OGD/R) without SeNPs or after 24 h pre-incubation with 0.5 and 2.5 µg/ml SeNPs. (**B**) Cytogram demonstrating the viability of cortical primary cultured neurons and astrocytes. X-axis—the intensity of PI fluorescence; Y-axis—the intensity of Hoechst 33342 fluorescence. Cells were stained with the probes 24 h after the 2 h OGD and 24 h reoxygenation depending on the presence of SeNPs. (**C**) Neuroprotective effects of SeNPs on the induction of necrosis and apoptosis after 2 h OGD and 24 h reoxygenation. The percentage of living cells (black column) and cells in which the processes of early apoptosis (violet column), apoptosis (green column), and necrosis (red column) were detected. Cell cultures were returned to CO_2_-incubator for 24 h after 2 h OGD. Each point represents the mean from an individual cell culture. At least 2000 cells were analyzed for each sample. N cell cultures = 5; n coverslips with cells for each sample = 15. (**D**) Hydrolysis of the fluorogenic substrate of caspase-3 (NucView-488) during a 40-min OGD treatment and the 90 min of reoxygenation, indicating apoptosis induction in neurons and astrocytes of cerebral cortex in the presence and absence of 0.5 µg/ml SeNPs. (**E**, **F**) Effect of 24-h incubation of cortical cells with 0.5 µg/ml SeNPs on expression of genes involved in regulation of apoptosis and necrosis (**E**), and encoding of selenoproteins (**F**) after 2 h OGD and 24 h reoxygenation. Dashed line—level of gene expression in controls (with 0.5 µg/ml SeNPs, but without OGD/R). The number of samples is 5 (cell cultures number = 5). Statistical significance was assessed using one-way ANOVA followed by the Tukey–Kramer test. n/s—data not significant (*p* > 0.05), *P < 0.05, **P < 0.01, and ***P < 0.001.

Since SeNPs (below 10 µg/ml) cause an increase in the expression of anti-apoptotic genes, but at the same time activate caspase-3 (except for 0.5 µg/ml), we proposed using two concentrations of SeNPs (0.5 and 2.5 µg/ml) as a neuroprotector in OGD/reoxygenation. For this, cortical cells were incubated for 24 h with SeNPs, and then subjected to 2-h OGD (oxygen–glucose deprivation, OGD) followed by 24-h reoxygenation, for which the cultures were returned to the CO_2_ incubator. In control cultures, 24 h after 2 h of OGD and 24 h of reoxygenation in most cells, increased fluorescence of HO342 and PI probes is recorded (Fig. 5A), which indicates cell death, by both type, necrosis and by the apoptosis (Fig. 5B). So, 24 h after OGD/R cell death occurs by the type of necrosis of 37 ± 4%, at the early and late stages of apoptosis 11 ± 4% and 51 ± 12% are recorded, respectively (Fig. 5C). However, pre-incubation of cell cultures with 0.5 and 2.5 μg/ml SeNPs (for 24 h) leads to complete disappearance of cells permeable to PI and a decrease in the number of cells with increased HO342 fluorescence (Fig. 5A, middle and bottom lines). This suggests inhibition of OGD/R-induced necrosis and suppression of late stages of apoptosis (Fig. 5B). The application of 0.5 µg/ml SeNPs leads to a complete suppression of OGD/R-induced necrosis; at the early and late stages of apoptosis, 14 ± 6% and 9 ± 4% of cells are recorded (Fig. 5C). A 24-h incubation of cortical cells with 2.5 μg/ml SeNPs leads to a similar effect, when after OGD/R necrosis is recorded only in single cells and early and late apoptosis in 13 ± 5% and 15 ± 6% of cells, respectively (Fig. 5C). The results of anti-apoptotic and anti-necrotic action of SeNPs are confirmed by staining cell cultures using the Apoptosis/Necrosis Detection Kit, when both concentrations of nanoparticles significantly increased cell survival after OGD/R (Supplementary, S1. Fig. 4). That is, 24-h incubation of cortical cells with selenium nanoparticles completely inhibits necrosis and significantly reduces apoptosis, which are caused by 2-h OGD, followed by 24-h reoxygenation.

As is known, in response to OGD and especially to reoxygenation, caspase-3 is activated and apoptosis is induced^37^. To investigate the protective effects of SeNPs, a series of experiments were carried out using the NucView-488. After detecting neurons and astrocytes in the field of view of the microscope, the fluorescence of NucView-488 was recorded in the cells under the action of OGD for 40 min and reoxygenation for 2 h (Fig. 5D). In response to OGD, in neurons caspase-3 activates after 10 ± 4 min with a fluorescence increase rate of 0.03 ± 0.002, but in response to reoxygenation, a repeated increase in caspase-3 production occurs (Fig. 5D, red curve) at a rate of 0.07 ± 0.002 (Table 2). In astrocytes, in response to OGD, caspase-3 is activated after a lag period of 30 ± 11 min (Fig. 5D, black curve) at a rate of 0.023 ± 0.002 (Table 2), and in response to reoxygenation, the rate of caspase-3 production increases to 0.04 ± 0.003 (Table 2). 24-h pre-incubation of cortical cells with 0.5 µg/ml SeNPs completely suppresses the process of caspase-3 activation during OGD in neurons and astrocytes (Fig. 5D, Table 2), and a lag phase of 18 ± 7 min is created during reoxygenation preceding the increase in fluorescence of NucView-488 (Fig. 5D). The rate of production of caspase-3 during reoxygenation with SeNPs in neurons is 0.06 ± 4.8^−4^ and in astrocytes 0.03 ± 4.8^−4^ (Table 2).

OGD for 2 h followed by 24-h reoxygenation leads to an increase in the expression of the gene encoding pro-apoptotic Bax by 2.3 times with an suppression of anti-apoptotic genes – Bcl-2, Bcl-xL, Socs3 by 59%, 65% and 73% , respectively (Fig. 5E). A more pronounced effect of OGD/R was found for the expression of genes encoding necrosis marker proteins. The expression of Eif2ak1, Rip1, Trail and Cas-1 is increased by 3, 5.8, 4.4 and 4.9 times, respectively (Fig. 5E). The neuroprotective effect of 0.5 µg/ml SeNPs is accompanied by an increase in the expression of anti-apoptotic genes—Bcl-2, Bcl-xL, Nrf2 by 4.5 times, 4.9, 2.3 and 2.83 times, respectively (Fig. 5E, red bars). The expression level of the pro-apoptotic Bax gene does not significantly change after SeNPs + OGD/R, but has a downward trend. The neuroprotective effect of 0.5 µg/ml SeNPs is accompanied by a decrease in the expression of necrotic markers, Eif2ak1 and Cas-1 by 68% and 77%, respectively (Fig. 5E).

As for the genes encoding the main selenoproteins, after OGD/R, there is an increase in the expression of SelK, SelN, and SelT by 390%, 62 and 87%, respectively (Fig. 5F). However, after pre-incubation with 0.5 µg/ml SeNPs and OGD/R, the expression of the SelK and SelT genes is also increased by 2.8 and 4.6 times (Fig. 5F). At the same time, the level of SelP expression is suppressed both after OGD/R and after incubation with selenium nanoparticles, close to 0, compared with intact cells (Fig. 5F).

Thus, pre-incubation of cortical cells with selenium nanoparticles protects them from damage during prolonged (2 h) and shorter (40 min) OGD, almost completely suppressing necrotic processes and significantly inhibiting apoptosis. In particular, both in neurons and astrocytes, after incubation with SeNPs, caspase-3 is completely inhibited during OGD, and caspase-3-induced apoptosis is suppressed several times. At the level of gene expression, SeNPs activate anti-apoptotic genes and suppress the expression of pro-apoptotic—Bax after OGD and reoxygenation, and also participate in the activation of selenoproteins involved in the mechanisms of ER-stress during OGD.

## Discussion

SeNPs are widely used in biomedical and pharmaceutical applications due to their antioxidant, antimicrobial, anti-diabetic and antitumor effects^44^. SeNPs can act as a pro- and antioxidant depending on their concentration and pathological process in the body^21,45^.

### Features of the action of SeNPs on primary cultured neurons and astrocytes under normal physiological conditions

Here, we tested the effect of SeNPs on brain cells at different concentrations under physiological conditions. The authors would like to emphasize that the results discussed at this stage, obtained under physiologically normal conditions, characterize the effect of SeNP primary cultured neurons and astrocytes under normal conditions before the ischemic model. It was found that high doses of SeNPs (2.5–10 µg/ml) dose-dependently lead to apoptosis, suppressing necrosis, which is accompanied by an increase in the activity of caspase 3, but at the same time there is an increase in the expression of anti-apoptotic genes Bcl-2, Bcl-xL, genes of redox homeostasis Nrf2 and Socs3 and suppression of expression of the pro-apoptotic marker Bax.

It has been shown that high doses of selenium can induce thiol/disulfide redox modification of many proteins, which can lead to their unfolding or misfolding in the ER (endoplasmic reticulum), causing stress to the ER^46^. Based on our results, it turns out that high concentrations of SeNPs, on the one hand, are toxic to cells, as they lead to cell death, but on the other hand, they modulate pro-apoptotic cascades in such a way that there is an increase in anti-apoptotic adaptation markers such as Bcl-2, Bcl- xL, Nrf2 and Socs3. Perhaps this is due to the activation of adaptive processes following the stress of the ER. This assumption has a basis, since we also observe changes in the expression of selenoproteins-resident ER under the action of SeNPs: a high concentration of SeNPs (5 μg/ml) causes an increase in the expression of SelK, SelN, SelT, SelP.

This assumption has a basis, since we also observe changes in the expression of selenoproteins-resident ER under the action of SeNPs: a high concentration of SeNPs (5 μg/ml) causes an increase in the expression of SelK, SelN, SelT, SelP. Probably at a high concentration of SeNPs selenium is absorbed by cells and is able to participate in the resynthesis of selenoproteins, increasing the level of their expression. It can be assumed that it is not the mitochondrial cascade that leads to dose-dependent apoptosis when exposed to high doses of SeNPs, since the anti-apoptotic proteins Bcl-2 and Bcl-xL block it, but pro-apoptotic signaling cascades associated specifically with ER stress. Pro-apoptic signaling cascades involving the UPR pathways with PERK, IRE1 can also lead to the activation of effector caspase-3 and cell death. Stimulation of apoptosis with large doses of SeNPs may have a positive effect in the case of oncological diseases, since SeNPs trigger a metabolic transition from uncontrolled cell death, necrosis, to a controlled pathway of cell death—apoptosis. This could have potential therapeutic value. Examples of the use of SeNPs as an anticancer agent are widely researched^21,39,47,48^.

Interestingly, astrocytes were especially sensitive to a high dose of SeNPs: when exposed to 5 µg/ml SeNPs, the rate of caspase-3 production was higher than in neurons. As a result, apoptosis was induced faster and stronger in astrocytes than in neurons. According to the literature, astrocytes are more stable and less vulnerable to oxidative stress than neurons. It has been shown that astrocytes die by delayed necrosis, demonstrating less apoptotic signaling^49,50^. Most neurons in co-cultures die after 60–90 min OGD while astrocytes are irreversibly damaged only after 4–6 h^51^. The viability of astrocytes during OGD persists longer than neurons^52^. Moreover apoptosis was induced in neurons with a higher rate as compared to astrocytes under the influence of classical inducer of apoptosis staurosporine on the brain cells culture.

Thus, we demonstrate for the first time that SeNPs modify the state of "resistance" of astrocytes to stressful conditions, triggering rapid apoptotic signals in them. This phenomenon can be associated with selenoproteins, since SeNPs are able to alter the expression of selenoproteins of ER residents which we also demonstrate. This is in good agreement with the literature data where it has been shown that during OGD astrocytes are able to increase the expression of antioxidant selenoproteins during brain injury^53^. Our assumption is confirmed by the dynamics of the increase in the activity of caspase-3. After the initial increase, we observe a plateau that lasts about 70 min, and then exponentially activity growth. Probably, protein synthesis occurs during the plateau, which leads to the rapid development of pro-apoptotic ER stress signals and activation of the caspase-cascade with the participation of caspase-3.

It was shown that astrocytes differ in the spectrum and the amount of selenoproteins in comparison with neurons. For example, SelS expression is noticeable in neurons and practically not found in astrocytes^54,55^. Expression of SelS is intensely activated in astrocytes in the case of a brain injury. Overexpression of SelS increases astrocyte resistance to ER stress and inflammatory stimuli. In contrast, suppression of SelS put at risk of the astrocytes viability^54^. In the brain, SelP is primarily expressed in astrocytes^56,57^. SelP is one of the most studied selenoproteins. It contains 10 selenocysteines, is responsible for the transport of selenium, especially for the retention of selenium in the brain, modulates selenium homeostasis and redox balance in the human brain^58^.

Of particular interest is the effect of a low dose of SeNPs—0.5 µg/ml on brain cells. Almost complete suppression of basic necrosis and apoptosis was observed. This was accompanied by a decrease in the expression of the pro-apoptotic protein, a decrease in the activity of caspase-3, a decrease in the level of ER selenoproteins, but at the same time an increase in the expression of antiapoptotic proteins. An increase in cell survival may indicate that low doses of SeNPs under normal physiological conditions improve the state of brain cells, and under unfavorable conditions can protect cells, i.e. this dose can be prophylactic or moreover provide preconditioning of brain cells, i.e. to prepare the metabolism and state of nerve cells in such a way that under stress (ischemia) the cells will more easily cope with the stressful situation. Probably, it also affects the processes associated with the participation of selenoproteins of ER residents—SelK, SelN, SelT, SelP. We observe a decrease in their expression level, which may indicate the activation of the adaptive UPR pathway through the IRE1 signaling pathway, in which IRE1 can degrade mRNA during RIDD^59,60^. There is evidence that preconditioning is an important phenomenon of CNS adaptation to subsequent ischemia, which is based on the redistribution of intracellular calcium reserves^61,62^.

### Neuroprotective action of SeNPs under OGD/reoxygenation conditions

Further in our work, we investigated the mechanisms of the protective effect of a low dose of SeNPs on brain cells during OGD/R. Ischemia-induced brain damage is largely mediated by oxidative stress, glutamate-induced excitotoxicity, mitochondrial dysfunction and endoplasmic reticulum stress, activation of pro-apoptotic transcription factors, ultimately leading to rapid death of brain cells by apoptosis^63^. An important molecular event that mediates the progressive activation of the pathways of cell death after stroke is the excessive amount of intracellular calcium, which is constantly maintained at a low level under physiological conditions. Impaired Ca^2+^ homeostasis activates either programmed cell death (apoptosis) or collapse due to loss of control over cellular mechanisms (necrosis)^2^. SeNPs may be involved in the restoration of calcium homeostasis. SeNPs have been shown to enhance the expression of parvalbumin in the brain and exert a neuroprotective effect through maintaining Ca^2+^ homeostasis^64,65^, which is in good agreement with our previous data^32^ and can suppress necrosis and inhibit apoptosis in OGD/R. The involvement of SeNPs in maintaining calcium homeostasis may be mediated by the ER selenoproteins, which will be discussed below.

One of the consequences of impaired calcium homeostasis is mitochondrial dysfunction during ischemia. Damage to mitochondria causes caspase-induced apoptosis. Mitochondrial apoptosis is mainly regulated by proteins of the B-cell lymphoma-2 (Bcl-2) family, which are pro-apoptotic (e.g. Bax, Bok) or anti-apoptotic (e.g. Bid, Bcl-2)^66^. It has been shown that Bcl-2 can play an important role in the suppression of apoptosis in neuronal cells after excitotoxic damage or ischemia^67^. This factor can also be activated within PI3K/Akt^68^. It is known that compounds containing selenium are able to suppress apoptosis through an increase in the level of Bcl-2 expression and suppression of Bax in the cells of the reproductive system^69,70^. But there is practically no data on the neuroprotective mechanisms of SeNPs on neurons in the cerebral cortex. The data on the antiapoptotic effect of selenium-containing compounds are in good agreement with our results on the enhancement of basic and OGD-induced expression of antiapoptotic genes (Bcl-2, Bcl-xL) against the background of suppression of Bax.

Along with mitochondria, stressed ER also induces the activation of caspase proteins, which leads to neuronal apoptosis^70^. It is known that ischemic stroke causes accumulation of misfolded proteins and loss of calcium homeostasis, which leads to impaired ER function and stress^45,71^. Currently, the role of selenoproteins in the regulation of ER stress is being actively studied, with selenoproteins localized mainly in the ER, which include SELK, SELN, SELS, SELM, SELT, SEP15, and DIO2, are of particular interest^72–75^. The described processes of ER stress development are consistent with our results. OGD promoted the activation of two pro-apoptotic signaling pathways UPR at once: PERK and IRE1 through the alternative branch IRE1/TRAF2/ASK1/JNK. This is confirmed by an increase in the level of mRNA of the protein marker of apoptosis Bax and a decrease in the expression of anti-apoptotic proteins Bcl-2, Bcl-xL and activation of caspase-3.

As described earlier, a low dose of SeNPs under physiological conditions led to a decrease in the level of expression of selenoprotein genes, and also in OGD/R, we observed a sharp decrease in SelP expression. Possibly, in the first case, the IRE1 signaling pathway of the UPR is triggered by a low dose of SeNPs, and in the second case, by ischemia-like conditions, while, through its endoribonuclease activity, IRE1 can degrade many mRNAs without a specific sample, including the mRNAs of the proteins we study, in a process known as regulated IRE1α-dependent decay (RIDD)^59,60^. Low doses of SeNPs lead to activation of the anti-apoptotic IRE1-XBP1 pathway, and possibly the Nrf-2/HO-1 antioxidant signaling pathway through PERK interaction with Nrf2, but also suppress the PERK/eIF2α/ATF4/CHOP-mediated ER stress level, thus thereby reducing apoptosis. This is confirmed by the increased expression of the ER selenoproteins, Bcl-2, Bcl-xL, and Nrf2.

SeNPs are considered a potential therapy for ischemic stroke. The anti-apoptotic effect of selenium and its nanosized particles has been demonstrated in various experimental protocols through the enhancement of anti-apoptotic markers and the suppression of pro-apoptotic markers^69,70^. It was also shown that 11-mercapto-1-undecanol (MUN) decorated SeNPs decreases cisplatin-induced nephrotoxicity in human kidney HK-2 proximal tubular cells through suppression of caspase-3 and ROS production^76^. Anti-apoptotic effect of selenum against excitotoxicity in rat striatum was shown to be mediated via IκB-α degradation, NF-κB nuclear translocation and caspase-3-like activation^77^, when in response to OGD/R complete inhibition of the first phase of caspase-3 activation is observed and a significant suppression of the rate of increase in the second phase after incubation with SeNPs. This also plays a significant role in neuroprotection and cell death protection and is consistent with our data.

There is other compelling evidence that SeNPs suppress OGD/R-induced necrosis. It is known that the toxicant acrylamide induces necrosis and apoptosis in the brain, and the addition of 0.5 μg/ml SeNPs to the drinking water of rats leads to a significant and more effective suppression of these pathological processes in the corpus striatum compared to ferulic acid^29^, which is also in good agreement with our data. It was shown that SeNPs with an increase in concentration mainly induce early stages of apoptosis without induction of necrosis on keratinocytes of the epidermis^78^. Similarly, in our experiments, an increase in the concentration of SeNPs leads to the induction of early apoptosis in the cells, which occurs against the background of complete suppression of the basic OGD/R-induced necrosis in vitro.

In our work we focused on ER selenoproteins, such as SelP, SelK, SelT, since ER stress plays an important role in the mechanisms of survival during OGD. We demonstrate that, under the action of OGD/R, an increase in the expression of SelK, SelN, and SelT occurs, while the level of SelP expression is significantly suppressed both after OGD/R and after incubation with selenium nanoparticles and is close to 0. After preliminary incubation of the brain cells with 0.5 µg/ml SeNPs, we observed a significant increase in the expression of the SelK and SelT genes. It turns out that these selenoproteins—SelK, SelN, SelT and SelP is most sensitive to ischemic conditions (especially SelT), as well as to the effects of SeNPs. This indicates their participation in signaling processes leading either to death or survival of brain cells, i.e. they possible provide the neuroprotective effect of SeNPs.

Noteworthy, is the significant decrease in SelP expression at 24-h incubation of cortical cells with 0.5 µg/ml SeNPs (Fig. 4B). SelP is unique in that it has 10 selenocysteine residues and is widely present in the brain. The main function of this protein is to transport and supply the brain with selenium. It serves as a "survival factor" for neurons in culture. SelP^−/−^ knockout mice are characterized by hypoplasia and die, while overexpression of this selenoprotein in hepatocytes, together with a Se containing diet leads to a significant cancellation of the knockout effect and contributes to the survival of mice^79,80^. Using immunocytochemical staining it was shown that SelP is predominantly localized in neurons and ependymal cells along the ventricles and less often in astrocytes^57^. But at the same time revealed that SelP is formed and secreted by cultured astrocytes in response to neuronal activation and accordingly astrocytes can serve as an intracerebral source of selenium for neurons controlling its availability^81,82^. A decrease in SelP expression 24 h after OGD/R in our experiments may indicate the inhibitory effect of OGD on this protein and selenium transport across the plasma membrane. On the other hand, there is no need for a specific transporter for the transport of SeNPs. It is also possible that during a stressful situation against the background of OGD the cell saves it’s metabolic and energy resources in order to restore cell homeostasis as quickly and painlessly as possible. We also previously suggested that SelP mRNA can be destroyed under ER stress.

An increase in the level of [Ca^2+^]_i_ in neurons caused by cAMP application significantly increases the level of SelT expression, as well as SelT overexpression affects the Ca^2+^ homeostasis of neurons^83^. It is known that under OGD conditions, a biphasic increase in [Ca^2+^]_i_ occurs^84^, which may affect the level of SelT expression. SelT shows significant growth 24 h after OGD/R and is greatly enhanced by OGD/R in the presence of SeNPs, which may also promote cell survival. In addition, increased SelT expression promotes protein folding and activates the development of ER stress along the adaptive pathway.

We also observed an increase in the level of SelK selenoprotein expression when exposed to SeNPs. The SelK protein has been ascribed a neuroprotective role through the maintenance of calcium homeostasis. It has been shown that SelK can interact with palmitoyltransferase (DHHC6) and regulate the flux of Ca^2+^ ions in the ER through IP_3_R palmitoylation and SelK overexpression increases [Ca^2+^]_i_ in microglia^85–87^. SelK mRNA levels were significantly increased upon exposure to SeNPs. This suggests that SelK plays an important role in the development of the adaptive pathway together with SelT at the initial stages of ER stress initiation.

## Conclusions

The mechanisms of the protective action of SeNPs affect the adaptive signaling cascades UPR—PERK and IREA1a with the participation of ER residents selenoproteins, in particular, selenoprotein K and T. Protective mechanism also involves proteins of the Bcl-2 family, the processes of restoration of calcium homeostasis, inhibition of mitochondrial and ER stress pathways, and leading to ultimately to inactivation of caspase-3 and inhibition of apoptosis.

## Supplementary Information

Supplementary Information.

## Data Availability

The data presented in this study are available on request from the corresponding author.

## Abbreviations

SeNPs: Selenium nanoparticles
OGD: Oxygen and glucose deprivation
R: Reoxygenation
Real-Time PCR: Real-time polymerase chain reaction
Bcl-2: (apoptosis regulator Bcl-2, anti-apoptotic)
Bcl-xL: (B-cell lymphoma-extra large, anti-apoptotic)
Bax: (Bcl-2-associated X protein, pro-apoptotic) – Members of the Bcl-2 family of proteins
SelK, SelN, SelT, SelP: Selenoproteins K, N, T, P
Nrf2: Nuclear factor erythroid-derived 2-like 2
SOCS3: Suppressor of cytokine signaling 3
STAT3: Signal transducer and activator of transcription 3
ERS: Endoplasmic reticulum stress
