# Supplementary material for: Features of the cytoprotective effect of selenium nanoparticles on primary cortical neurons and astrocytes during oxygen–glucose deprivation and reoxygenation
Source: Sci Rep. 2022 Feb 2;12:1710. doi: 10.1038/s41598-022-05674-1 (PMC8810781; doi:10.1038/s41598-022-05674-1)
Supplement: Supplementary file 1 — Supplementary Information. [file 41598_2022_5674_MOESM1_ESM.docx]

| 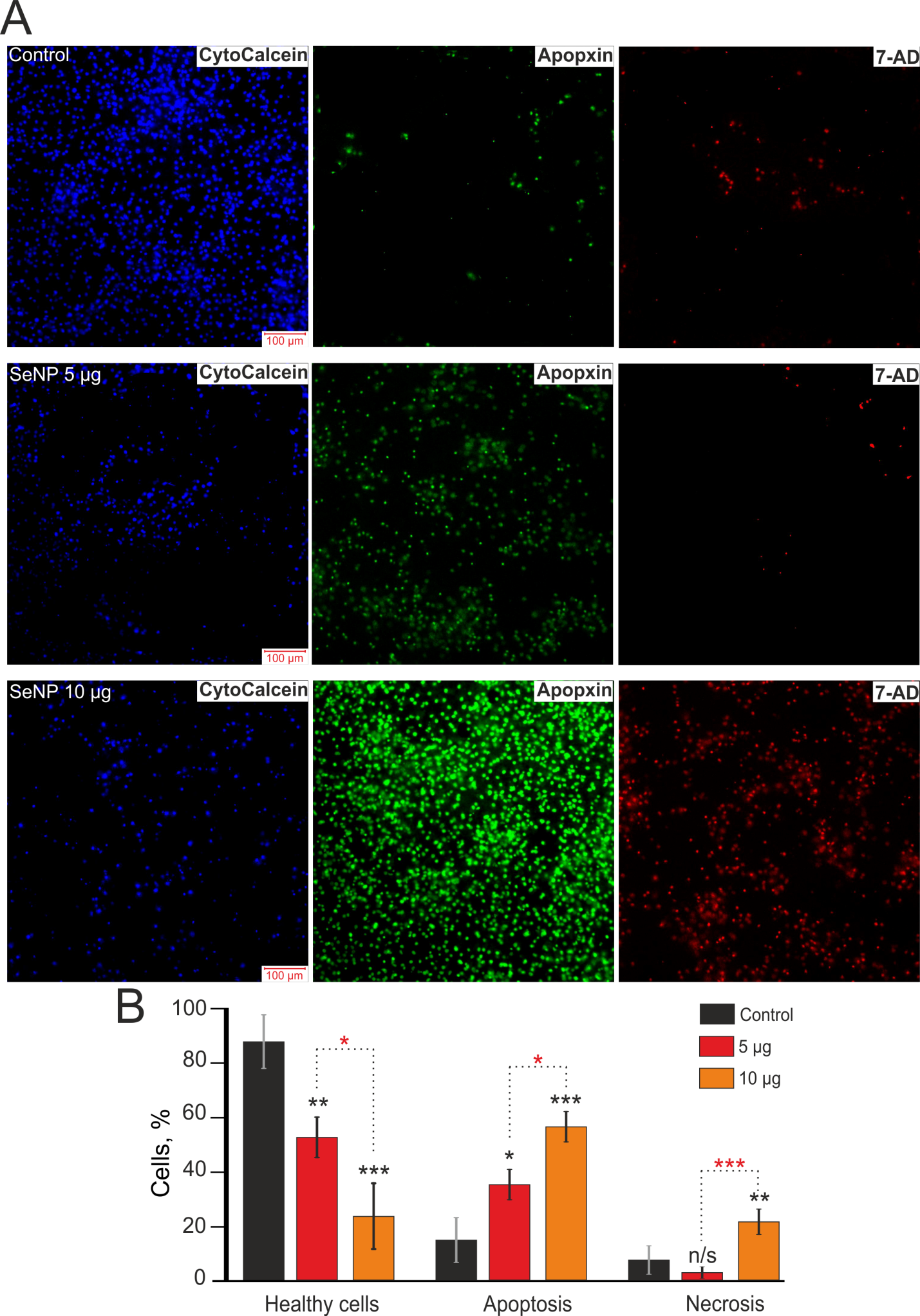 |
| --- |
| **S1. Figure 1.** Effect of pre-incubation of cortical primary cultured neurons and astrocytes with 5 µg and 10 µg SeNPs for 24 hours (induction of apoptosis and necrosis).  **A** – images of cell cultures stained with CytoCalcein - living cells indicator, Apopxin – apoptotic cells indicator and 7-AD (7-aminoactinomycin D) – necrotic cells indicator.  **B** – Cell survival in culture after pre-incubation with different concentrations of SeNPs. Shown are the results obtained on 3 cell cultures (N), n coverslips = 9, as mean ± SEM. Statistical significance was assessed using paired t-test. n/s – data not significant (p> 0.05), * P <0.05, ** P <0.01, and *** P <0.001. |

| 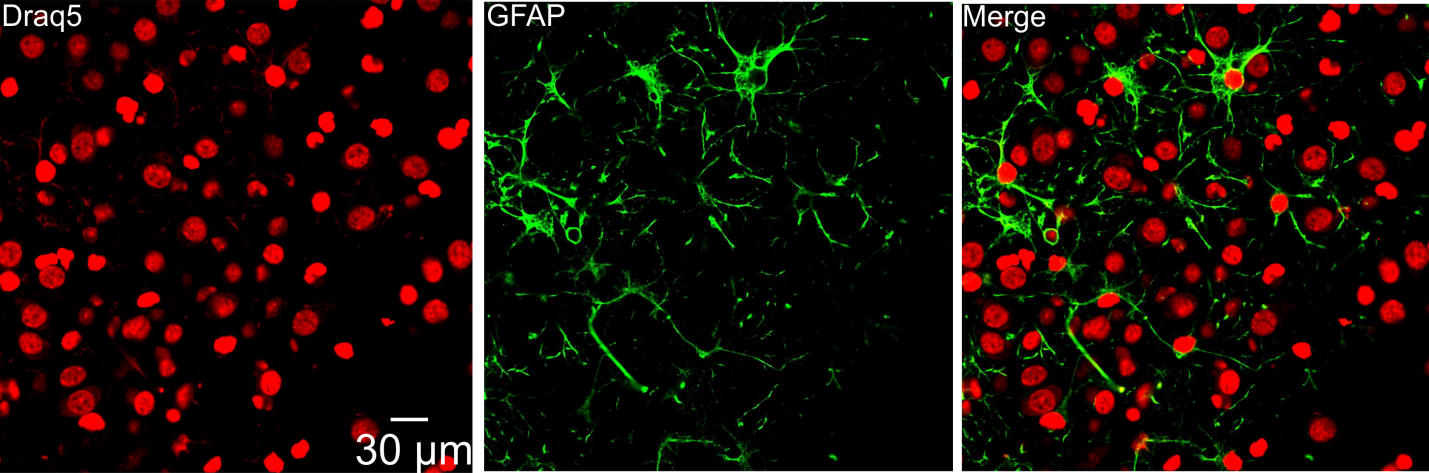 |
| --- |
| **S1. Figure 2.** Immunocytochemical staining of cortical cell culture with astrocytic marker, antibodies against glial fibrillary acidic protein (GFAP). The nuclei of all cells stained with Draq5 are shown in red. |


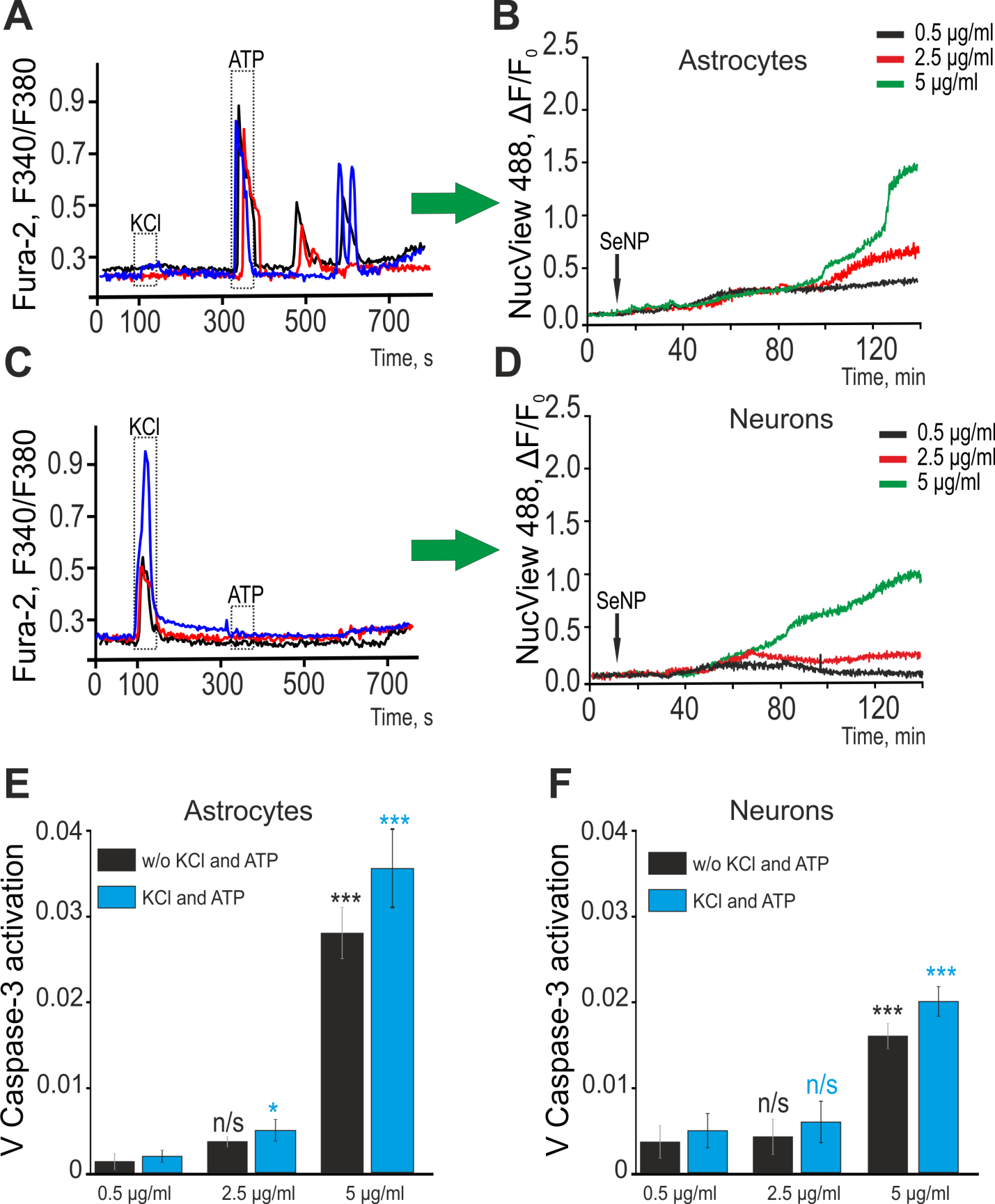


**S1. Figure 3.** Short-term application of KCl and ATP for the detection of neurons and astrocytes, respectively, does not affect the SeNPs-induced activation of caspase-3.

**A**, **C** – Registration of [Ca^2+^]_i_ dynamics using the Fura-2 fluorescent probe in astrocytes (**A**) and neurons (**C**). In response to a short-term (30 s) application of 35 mM KCl, neurons respond with Ca^2+^ signals, and astrocytes respond to the addition of 10 µM ATP. After registration of [Ca^2+^]_i_ dynamics without changing the field of view of the microscope, the excitation and registration filters for the NucView-488 fluorescent probe were replaced. Shown are the averaged Ca^2+^ signals obtained from several tens of cells.

**B**, **D** – Registration of caspase-3 activation by an increase in NucView-488 fluorescence in response to the addition of various concentrations of SeNPs in astrocytes (**B**) and neurons (**D**). Shown are the results obtained on 3 cell cultures (N), n coverslips = 3, as mean ± SEM.

**E**, **F** – The rate of caspase-3 activation in astrocytes (**E**) and neurons (**F**) with the application of various concentrations of SeNPs. Black columns – experiments on registration of NucView-488 fluorescence were performed without preliminary short-term application of KCl and ATP, and blue columns – after short-term application of KCl and ATP. The experiments shown in panels **A**–**D** and the rates of caspase-3 activation (**E**, **F**, blue bars) are blinded experiments. For black columns N = 3, n = 9 and for blue columns N = 3, n = 3. Statistical significance was assessed using one-way ANOVA followed by the Tukey-Kramer test. Comparison of experimental groups relative to control: n/s – data not significant (p> 0.05), * p <0.05 and *** p <0.001. The differences between blinded and not blinded experiments (black and blue bars) are not significant.

| 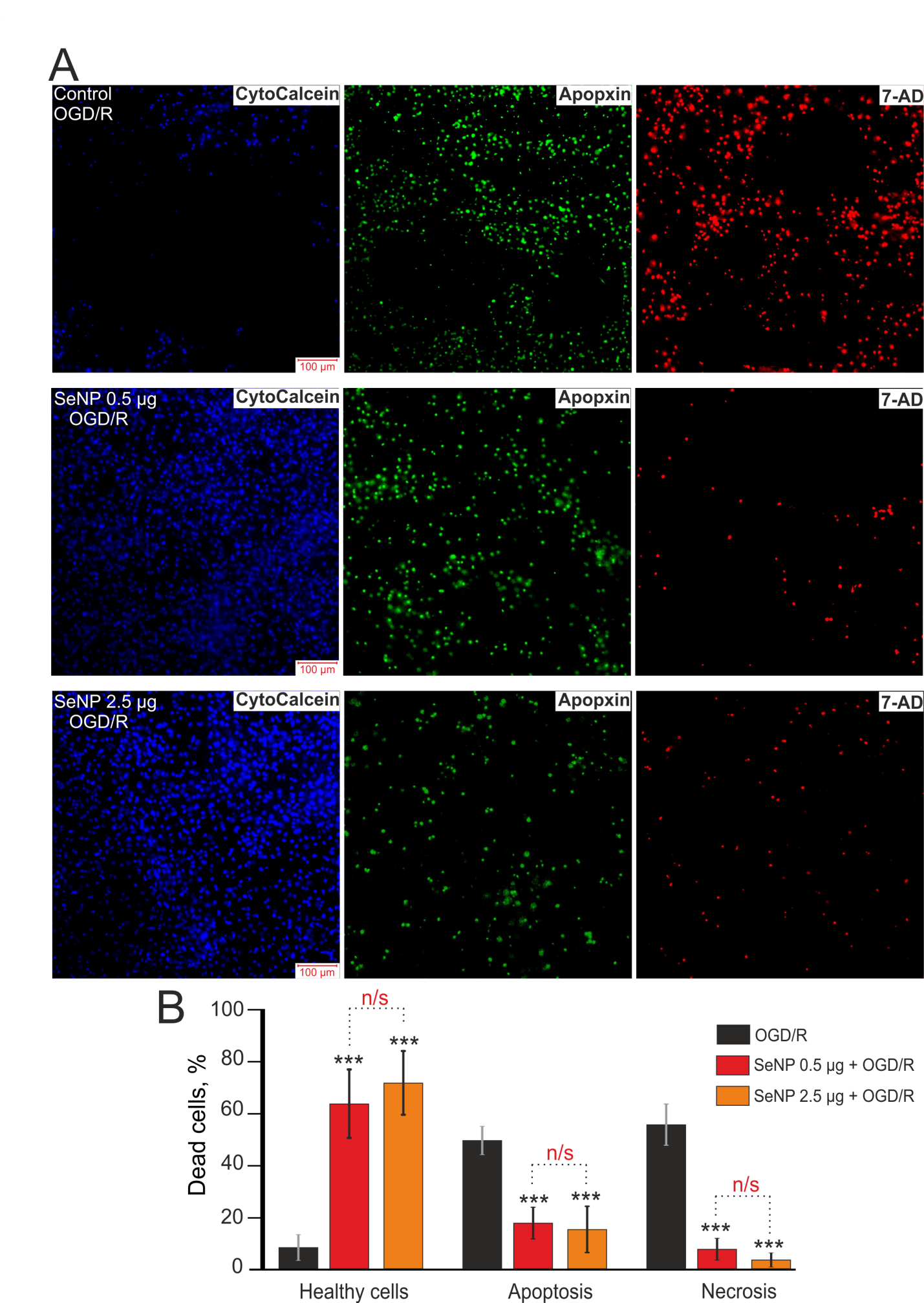 |
| --- |
| **S1. Figure 4.** Cytoprotective effect of 24-hour pre-incubation of cortical primary cultured neurons and astrocytes with 0.5 µg and 2.5 µg SeNPs under the action of OGD/R.  **A** – images of cell cultures stained with CytoCalcein - living cells indicator, Apopxin – apoptotic cells indicator and 7-AD (7-aminoactinomycin D) - necrotic cells indicator.  **B** – Cell survival in culture after preincubation with various concentrations of SeNPs and OGD/R. Shown are the results obtained on 3 cell cultures (N), n coverslips = 9, as mean ± SEM. Statistical significance was assessed using paired t-test. n/s – data not significant (p> 0.05), *** p <0.001. |
